# Supplementary material for: Unexpected Gene-Flow in Urban Environments: The Example of the European Hedgehog
Source: Animals (Basel). 2020 Dec 7;10(12):2315. doi: 10.3390/ani10122315 (PMC7762246; doi:10.3390/ani10122315)
Supplement: Supplementary file 1 [file animals-10-02315-s001.pdf]

Supplementary material

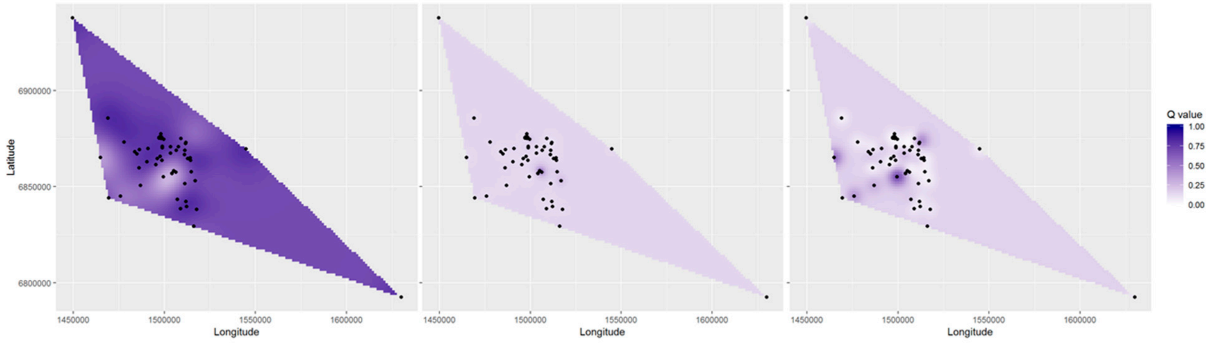

Figure S1
Map of all sampling locations (*N* = 143), displaying the distribution of individual genotype Q-values (STRUCTURE analysis results), values in-between are interpolated. Left: data for cluster 1, center: data for cluster 2, right: data for cluster 3. Plotting of results was carried out using the plot function in R.

Table S1 Origin of samples (*N* = 143)

ID	Year	Month	Address	lat_y	long_x	Source
110	2016	May	Trainierbahn Hoppegarten	52.4778846	13.6271494	IZW
127	2013	September	Rehwiese, Gerkrathstraße 2	52.42811	13.19973	IZW
128	2013	October	Moldaustr. 24, 10319 Berlin	52.49873	13.51299	Veterinary Practice
129	2013	October	Rohrwallallee 10, 12527 Berlin	52.39525	13.63471	Veterinary Practice
135	2013	October	Korkedamm 73, 12524 Berlin	52.4245	13.539	Veterinary Practice
136	2013	October	Moldaustr. 24, 10319 Berlin	52.49873	13.51299	Veterinary Practice
137	2013	September	Kablower Weg 89, 12526 Berlin	52.4034709	13.5853995	Veterinary Practice
138	2013	September	Kablower Weg 89, 12526 Berlin	52.4034709	13.5853995	Veterinary Practice

139	2013	September	Falkenberger Krugwiesen, 13057 Berlin	52.5641643	13.5338813	IZW
140	2013	September	Falkenberger Krugwiesen, 13057 Berlin	52.5641643	13.5338813	IZW
184	2016	July	Freischuetzstr., 13129 Berlin	52.6023802	13.45136	Veterinary Practice
185	2016	July	Gutenfelsstr. 14, 13129 Berlin	52.6005441	13.4493462	Veterinary Practice
186	2016	August	Gutenfelsstr. 14, 13129 Berlin	52.6005441	13.4493462	Veterinary Practice
187	2016	August	Gutenfelsstr. 14, 13129 Berlin	52.6005441	13.4493462	Veterinary Practice
188	2016	August	Schwarzelfenweg 19, 13088 Berlin	52.5714	13.46435	Veterinary Practice
189	2016	August	Strasse 7, 13129 Berlin	52.5983843	13.4486779	Veterinary Practice
191	2016	September	Strasse 26 Nr. 30, 13129 Berlin	52.595897	13.4727698	Veterinary Practice
192	2016	September	Urbacher Str., 13129 Berlin	52.6016518	13.4608262	Veterinary Practice
193	2016	October	Jungbornstr., 13129 Berlin	52.598014	13.4593083	Veterinary Practice
125	2013	September	Riesserseestr. 10, 12527 Berlin	52.4185357	13.5818458	Veterinary Practice
126	2013	September	Moldaustr. 30, 10319 Berlin	52.49834	13.51277	Veterinary Practice
134	2013	August	Volkspark Prenzlauerberg, Berlin	52.53564	13.4634372	IZW
176	2016	October	Eisenhuettenstadt	52.1436615	14.6419022	Hedgehog Station
179	2016	October	Eisenhuettenstadt	52.1436615	14.6419022	Hedgehog Station
182	2016	October	Zehlendorf, Berlin	52.4339586	13.2589089	Hedgehog Station
194	2016	October	Schwarzwaldstr./Ilsenstr.	52.598586	13.453231	Veterinary

			, 13129 Berlin			Practice
196	2016	October	Schwarzwaldstr., 13129 Berlin	52.598586	13.453231	Veterinary Practice
197	2016	November	Krontalerstr., 13125 Berlin	52.6112819	13.4577434	Veterinary Practice
198	2016	October	Gutenfelsstr. 14, 13129 Berlin	52.6005441	13.4493462	Veterinary Practice
199	2016	October	Hellersdorf,Berlin	52.536107	13.6049726	Veterinary Practice
200	2016	October	Gutenfelsstr. 14, 13129 Berlin	52.6005441	13.4493462	Veterinary Practice
203	2016	October	Freischuetzstr., 13129 Berlin	52.6023802	13.45136	Veterinary Practice
114	2017	April	Togostr. 45, 13351 Berlin	52.55369	13.33934	Veterinary Practice
116	2017	March	Alt-Tegel 47c, 13507 Berlin	52.58759	13.27552	Veterinary Practice
117	2017	May	Aroser Allee 111, 13407 Berlin	52.56659	13.35125	Veterinary Practice
119	2017	May	Ghanastr. 27, 13351 Berlin	52.56047	13.32984	Veterinary Practice
120	2017	July	Altglienike Feldweg	52.3975145	13.5554986	Veterinary Practice
141	2017	May	Tierpark, Berlin	52.5023038	13.5313559	IZW
142	2017	July	Volkspark Prenzlauerberg, Berlin	52.53564	13.4634372	IZW
143	2017	June	Tiergarten, Berlin	52.5144898	13.3500906	IZW
144	2017	May	Tierpark, Berlin	52.5023038	13.5313559	IZW
146	2017	May	Tierpark, Berlin	52.5023038	13.5313559	IZW
147	2017	June	Tiergarten, Berlin	52.5144898	13.3500906	IZW
149	2017	May	Tierpark, Berlin	52.5023038	13.5313559	IZW

150	2017	June	Buergerpark Pankow-Berlin	52.5694584	13.394732	IZW
152	2017	June	Tiergarten, Berlin	52.5144898	13.3500906	IZW
153	2017	June	Volkspark Prenzlauerberg, Berlin	52.53564	13.4634372	IZW
154	2017	May	Tierpark, Berlin	52.5023038	13.5313559	IZW
156	2017	June	Buergerpark Pankow-Berlin	52.5694584	13.394732	IZW
157	2017	NA	Treptower Park	52.48846	13.46974	IZW
158	2017	July	Hans-Baluschek-Park, 10829 Berlin	52.4644359	13.3570684	IZW
159	2017	July	Prenzlauerberg	52.54114	13.44009	IZW
161	2017	July	Hans-Baluschek-Park, 10829 Berlin	52.4644359	13.3570684	IZW
165	2017	May	Tierpark, Berlin	52.5023038	13.5313559	IZW
166	2017	May	Tiergarten, Berlin	52.5144898	13.3500906	IZW
167	2017	June	Tiergarten, Berlin	52.5144898	13.3500906	IZW
168	2017	July	Volkspark Prenzlauerberg, Berlin	52.53564	13.4634372	IZW
169	2017	July	Hans-Baluschek-Park, 10829 Berlin	52.4644359	13.3570684	IZW
170	2017	June	Volkspark Prenzlauerberg, Berlin	52.53564	13.4634372	IZW
172	2017	July	Volkspark Prenzlauerberg, Berlin	52.53564	13.4634372	IZW
174	2017	May	Tierpark, Berlin	52.5023038	13.5313559	IZW
175	2017	July	Volkspark Prenzlauerberg, Berlin	52.53564	13.4634372	IZW
305	2017	August	Tierpark, Berlin	52.5023038	13.5313559	IZW

306	2017	August	Tierpark, Berlin	52.5023038	13.5313559	IZW
307	2017	August	Tierpark, Berlin	52.5023038	13.5313559	IZW
308	2017	August	Tierpark, Berlin	52.5023038	13.5313559	IZW
311	2017	August	Tierpark, Berlin	52.5023038	13.5313559	IZW
312	2017	August	Tierpark, Berlin	52.5023038	13.5313559	IZW
314	2017	August	Tierpark, Berlin	52.5023038	13.5313559	IZW
315	2017	August	Tierpark, Berlin	52.5023038	13.5313559	IZW
317	2017	August	Tierpark, Berlin	52.5023038	13.5313559	IZW
318	2017	August	Tierpark, Berlin	52.5023038	13.5313559	IZW
319	2017	August	Tierpark, Berlin	52.5023038	13.5313559	IZW
320	2017	August	Tierpark, Berlin	52.5023038	13.5313559	IZW
321	2017	August	Tierpark, Berlin	52.5023038	13.5313559	IZW
322	2017	August	Tierpark, Berlin	52.5023038	13.5313559	IZW
324	2017	September	Eisenacher Str.,12629 Berlin	52.5402405	13.5902187	Pound
326	2017	October	Zum Erlenbruch, 15344 Strausberg	52.5680305	13.8773122	Pound
328	2017	September	Warnemünder Str. 18, 13059 Berlin	52.57423	13.50583	Pound
329	2017	September	Warnemünder Str. 18, 13059 Berlin	52.57423	13.50583	Pound
330	2017	September	Warnemünder Str. 18, 13059 Berlin	52.57423	13.50583	Pound
333	2017	September	12623 Berlin	52.5032943	13.6073142	Pound

334	2017	September	12624 Berlin	52.5032943	13.6073142	Pound
335	2017	September	12625 Berlin	52.5032943	13.6073142	Pound
337	2017	October	KGA Märchenland, 13089 Berlin	52.5749069	13.4650705	Pound
338	2017	November	Belziger Ring 36, 12689 Berlin	52.56367	13.57531	Pound
340	2017	October	Mahlerstraße, 13088 Berlin	52.5468372	13.4537713	Pound
341	2017	October	Kleingartenanlage 750 Jahre Berlin, 13057 Berlin	52.5739373	13.5438938	Pound
342	2017	September	Tierpark, Berlin	52.5023038	13.5313559	IZW
344	2017	September	IZW Garten, Berlin	52.50597	13.52148	IZW
345	2017	October	Treptower Park	52.48846	13.46974	IZW
346	2017	August	Treptower Park	52.48846	13.46974	IZW
348	2017	August	Treptower Park	52.48846	13.46974	IZW
349	2017	August	Treptower Park	52.48846	13.46974	IZW
350	2017	August	Treptower Park	52.48846	13.46974	IZW
113	2017	April	Choise-le-Roi-Str. 3, Berlin	52.65611	13.19784	Veterinary Practice
118	2017	April	Vielitzsee OT Strubensee, 16835	52.9397147	13.021689	Veterinary Practice
309	2017	August	Nordbahnhof	52.5318835	13.3883826	IZW
310	2017	August	Tierpark, Berlin	52.5023038	13.5313559	IZW
313	2017	August	Tierpark, Berlin	52.5023038	13.5313559	IZW
343	2017	September	Tierpark, Berlin	52.5023038	13.5313559	IZW
220	2017	April	Friedenstr., Berlin ?	52.5231715	13.4339728	Pound
231	2016	October	Friedenstr. 8, 16356 Ahrensfelde	52.58345	13.57986	Pound

235	2016	August	Glasberger Str. 43, 12555 Berlin	52.46971	13.57587	Pound
241	2016	September	Glambecker Ring 4, 12679 Berlin	52.55203	13.57497	Pound
243	2016	April	Zeuthen	52.3476518	13.6207615	Pound
248	2016	September	13053 Berlin	52.5559059	13.5055018	Pound
252	2016	October	Friedenstr., 16356 Ahrensfelde	52.5870868	13.5811324	Pound
257	2017	June	Dietrichstr. 5, 16356 Ahrensfelde	52.5975636	13.5557275	Pound
261	2016	September	Wolfshofstr. 25, 13591 Berlin	52.54398	13.1606	Veterinary Practice
300	2017	September	Kastanienallee 122/126, 12627 Berlin-Hellersdorf	52.543	13.60164	Pound
A1.317	2017	May	Treptower Park	52.4884599	13.4697445	IZW
A10.028	2016	July	Treptower Park	52.4884599	13.4697445	IZW
A11.028	2016	July	Treptower Park	52.4884599	13.4697445	IZW
A12.028	2016	July	Treptower Park	52.4884599	13.4697445	IZW
A13.028	2016	July	Treptower Park	52.4884599	13.4697445	IZW
A14.028	2016	July	Treptower Park	52.4884599	13.4697445	IZW
A15.028	2016	July	Treptower Park	52.4884599	13.4697445	IZW
A16.028	2016	July	Treptower Park	52.4884599	13.4697445	IZW
A2.317	2017	June	Treptower Park	52.4884599	13.4697445	IZW
A20.038	2016	July	Treptower Park	52.4884599	13.4697445	IZW
A21.038	2016	July	Treptower Park	52.4884599	13.4697445	IZW

A22.038	2016	July	Treptower Park	52.4884599	13.4697445	IZW
A23.038	2016	August	Treptower Park	52.4884599	13.4697445	IZW
A27.078	2016	June	Treptower Park	52.4884599	13.4697445	IZW
A28.078	2016	June	Treptower Park	52.4884599	13.4697445	IZW
A3.317	2017	July	Treptower Park	52.4884599	13.4697445	IZW
A30.078	2016	June	Treptower Park	52.4884599	13.4697445	IZW
A31.078	2016	June	Treptower Park	52.4884599	13.4697445	IZW
A32.078	2016	June	Treptower Park	52.4884599	13.4697445	IZW
A34.078	2016	June	Treptower Park	52.4884599	13.4697445	IZW
A35.088	2016	July	Treptower Park	52.4884599	13.4697445	IZW
A37.088	2016	July	Treptower Park	52.4884599	13.4697445	IZW
A4.317	2017	June	Treptower Park	52.4884599	13.4697445	IZW
A43.088	2016	October	Treptower Park	52.4884599	13.4697445	IZW
A47.098	2015	August	Treptower Park	52.4884599	13.4697445	IZW
A5.317	2017	June	Treptower Park	52.4884599	13.4697445	IZW
A56.098	2015	September	Treptower Park	52.4884599	13.4697445	IZW
A59.108	2015	August	Treptower Park	52.4884599	13.4697445	IZW
A61.108	2015	August	Treptower Park	52.4884599	13.4697445	IZW
A62.108	2015	August	Treptower Park	52.4884599	13.4697445	IZW

A68.108	2015	September	Treptower Park	52.4884599	13.4697445	IZW
A9.028	2016	July	Treptower Park	52.4884599	13.4697445	IZW

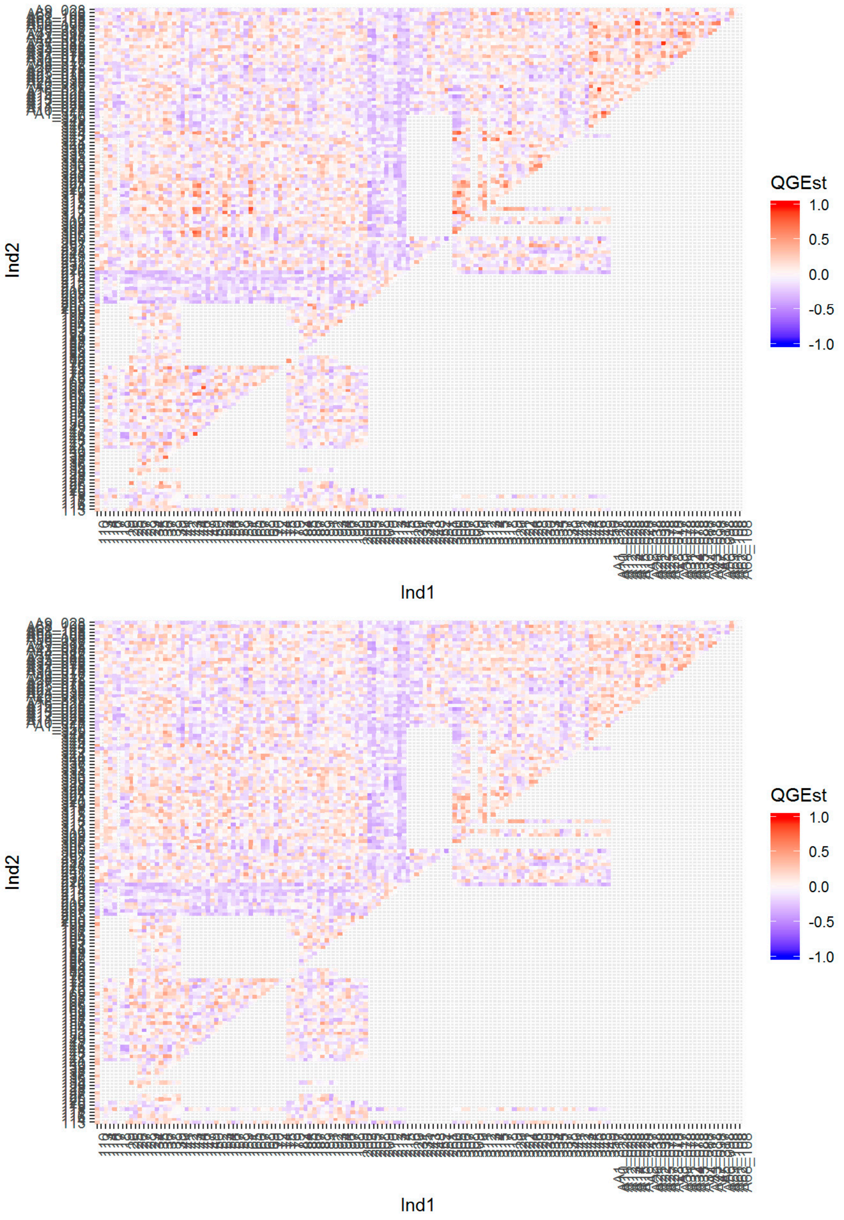

Figure S2 Pairwise relatedness after Queller and Goodman (QGEst) of sampled genotypes (Ind1 and Ind2) before (left) and after (right) removing related genotypes r > 0.5(darker red)

Table S2

Unique genotypes ($N = 143$) of hedgehogs in Berlin across ten microsatellite loci

ID	EEU1		EEU2		EEU3		EEU4		EEU5		EEU6		EEU12H		EEU36H	
110	135	139	257	257	145	153	148	148	113	129	145	145	91	97	246	[illegible]
127	135	135	257	257	145	163	160	160	113	135	145	145	95	97	240	[illegible]
128	129	129	259	267	149	165	148	160	115	129	145	145	97	97	246	[illegible]
129	135	143	259	267	149	163	160	170	113	135	145	155	91	97	236	[illegible]
135	135	139	259	279	149	153	156	158	113	113	145	145	91	91	246	[illegible]
136	129	139	267	269	145	165	148	148	115	129	145	145	91	97	246	[illegible]
137	135	135	261	267	131	163	158	160	109	113	145	145	91	91	248	[illegible]
138	135	139	261	273	163	163	158	160	109	113	145	145	91	91	248	[illegible]
139	131	135	269	269	145	155	144	158	109	113	145	145	91	97	240	[illegible]
140	135	139	267	281	145	153	148	148	115	129	145	145	91	97	240	[illegible]
184	129	131	267	269	145	145	154	160	109	129	145	145	91	91	240	[illegible]
185	135	137	269	271	153	169	146	152	109	113	145	159	91	97	256	[illegible]
186	139	139	265	267	155	163	148	152	113	127	145	145	97	97	256	[illegible]
187	137	139	257	273	149	169	148	164	113	115	145	159	91	97	238	[illegible]
188	129	139	257	269	153	179	160	170	113	129	145	145	91	95	240	[illegible]
189	135	135	267	267	153	153	148	156	113	113	145	155	91	91	240	[illegible]
191	129	129	267	267	153	169	152	152	113	113	145	145	91	97	256	[illegible]
192	135	139	257	259	159	179	146	160	107	113	145	147	91	97	246	[illegible]
193	129	139	269	277	169	169	160	164	113	113	145	145	91	97	240	[illegible]
125	129	139	267	269	153	163	148	150	113	115	145	145	91	91	240	[illegible]
126	129	129	267	269	145	163	148	148	113	115	145	159	91	91	240	[illegible]
134	135	139	267	275	149	163	148	150	113	115	145	145	95	97	240	[illegible]
176	131	135	259	259	153	159	148	164	113	113	145	157	91	97	244	[illegible]
179	131	131	259	259	153	153	148	150	113	113	145	147	91	97	244	[illegible]
182	129	139	267	279	153	153	160	160	107	135	145	145	91	91	246	[illegible]
194	139	139	257	267	145	159	148	160	113	123	145	145	97	97	240	[illegible]
196	139	139	259	267	145	179	148	160	107	123	145	145	91	97	238	[illegible]
197	131	139	269	277	153	163	148	158	107	113	145	155	91	91	248	[illegible]
198	129	137	259	267	163	165	148	158	109	113	145	145	97	97	248	[illegible]
199	131	131	257	257	153	153	148	148	113	125	145	145	91	91	250	[illegible]

200	129	137	269	273	149	169	148	154	107	115	145	145	97	97	240	[illegible]
203	137	139	265	273	149	163	148	148	113	127	145	145	97	97	248	[illegible]
114	131	139	259	269	149	153	158	160	113	113	145	145	91	97	240	[illegible]
116	135	135	263	265	159	177	148	160	113	129	145	145	95	97	238	[illegible]
117	135	139	267	271	145	169	150	170	113	123	145	145	97	97	248	[illegible]
119	131	139	263	273	145	149	152	160	113	139	145	145	91	95	250	[illegible]
120	135	139	269	281	149	169	148	160	115	139	145	147	91	91	236	[illegible]
141	129	139	257	263	153	163	148	158	121	129	145	155	91	97	246	[illegible]
142	135	135	263	273	153	163	148	158	113	115	145	145	91	95	240	[illegible]
143	129	129	257	259	153	159	150	150	109	113	145	145	91	97	240	[illegible]
144	129	129	257	267	153	179	148	158	113	129	145	155	97	97	246	[illegible]
146	129	129	257	267	153	179	148	148	113	129	145	155	97	97	240	[illegible]
147	139	139	257	271	131	159	156	170	115	115	145	155	91	97	278	[illegible]
149	129	139	271	271	153	153	158	160	113	129	145	145	93	95	246	[illegible]
150	131	135	257	265	159	163	158	164	115	121	145	145	97	97	256	[illegible]
152	139	139	269	269	153	153	148	148	113	113	145	145	97	97	240	[illegible]
153	135	139	267	269	145	163	156	158	113	113	145	155	91	95	240	[illegible]
154	129	139	265	267	163	179	148	160	113	113	145	155	91	97	246	[illegible]
156	135	139	267	267	153	163	148	164	109	113	145	145	91	97	240	[illegible]
157	129	131	259	267	145	179	158	158	115	115	145	153	91	95	248	[illegible]
158	135	135	273	275	149	165	166	170	113	113	145	155	95	97	248	[illegible]
159	135	139	275	275	149	165	148	160	115	129	145	145	91	91	248	[illegible]
161	129	139	267	279	149	153	158	158	113	113	145	155	91	91	236	[illegible]
165	129	129	257	265	153	163	158	160	113	129	145	145	97	97	246	[illegible]
166	139	139	269	273	153	163	148	148	113	113	155	155	91	91	240	[illegible]
167	139	139	257	271	131	175	170	170	113	115	145	155	97	97	278	[illegible]
168	139	139	267	269	145	165	158	158	113	115	145	155	91	91	236	[illegible]
169	131	135	257	257	145	145	158	170	113	121	145	145	91	91	248	[illegible]
170	131	135	269	269	145	153	158	160	113	113	145	155	95	97	248	[illegible]
172	135	139	261	269	145	163	150	160	115	129	145	147	91	95	240	[illegible]
174	129	129	267	281	145	153	148	148	113	129	145	155	91	97	240	[illegible]
175	135	139	261	273	153	163	158	170	113	115	145	155	91	91	248	[illegible]
305	129	139	257	265	153	163	148	152	113	113	145	155	97	97	246	[illegible]

306	129	129	257	271	153	153	148	148	113	129	145	155	97	97	246	[illegible]
307	129	129	265	273	159	163	148	160	113	113	145	145	97	97	240	[illegible]
308	131	135	261	273	145	159	148	158	113	129	145	145	91	97	240	[illegible]
311	135	135	267	273	159	181	144	148	113	113	145	145	91	97	240	[illegible]
312	129	139	257	265	153	163	148	160	113	113	145	155	97	97	246	[illegible]
314	131	139	261	265	163	163	148	160	113	113	145	145	91	97	246	[illegible]
315	135	139	265	267	163	163	158	158	113	113	145	145	91	97	246	[illegible]
317	131	139	267	267	165	165	148	160	113	129	145	145	91	97	240	[illegible]
318	129	135	265	267	163	179	146	148	113	129	145	155	97	97	246	[illegible]
319	129	131	257	271	153	153	148	158	113	129	145	145	95	97	246	[illegible]
320	129	129	257	263	153	163	148	160	113	115	145	145	91	91	246	[illegible]
321	129	135	267	273	159	179	148	148	113	129	145	155	97	97	240	[illegible]
322	129	135	261	265	145	163	158	160	113	129	145	145	97	97	240	[illegible]
324	135	139	271	273	149	163	148	160	113	115	145	147	91	97	240	[illegible]
326	139	139	267	267	131	145	148	160	113	129	145	145	91	91	240	[illegible]
328	135	139	257	267	149	153	148	150	113	113	145	145	91	95	248	[illegible]
329	139	139	267	267	153	153	150	160	113	115	145	145	91	91	240	[illegible]
330	135	139	0	0	149	153	150	150	113	115	145	145	91	91	240	[illegible]
333	139	141	257	267	153	163	148	160	113	129	145	145	91	97	240	[illegible]
334	133	133	265	271	145	159	148	170	113	113	145	145	91	97	256	[illegible]
335	135	139	267	267	153	159	148	160	113	113	145	145	91	95	240	[illegible]
337	139	139	257	259	153	159	148	158	107	113	145	159	95	95	240	[illegible]
338	131	143	259	265	145	153	150	158	113	115	145	145	91	97	240	[illegible]
340	139	139	267	267	163	165	148	158	113	115	145	155	91	95	248	[illegible]
341	133	139	257	269	145	171	158	164	107	113	145	145	91	97	240	[illegible]
342	139	139	257	265	153	163	152	160	113	113	145	145	91	97	246	[illegible]
344	129	131	257	257	153	153	148	148	113	113	145	155	95	97	246	[illegible]
345	131	135	259	273	165	173	158	160	113	115	145	145	91	97	246	[illegible]
346	131	135	273	273	173	173	160	160	109	113	145	155	91	95	256	[illegible]
348	133	139	269	269	165	173	160	160	107	123	145	145	91	91	248	[illegible]
349	129	135	267	273	145	149	158	160	107	113	145	147	91	93	248	[illegible]
350	129	131	267	273	145	173	160	160	113	113	145	153	93	95	256	[illegible]
113	135	139	257	269	153	171	148	160	113	131	145	145	91	91	244	[illegible]

118	133	139	257	267	145	145	160	164	113	129	145	155	97	97	240	[illegible]
309	135	139	267	271	145	153	160	160	113	123	145	145	91	91	256	[illegible]
310	129	135	269	273	145	163	148	160	113	113	145	145	91	97	248	[illegible]
313	129	129	257	267	153	179	158	160	129	129	145	145	97	97	246	[illegible]
343	129	131	267	267	131	179	156	158	115	115	145	145	91	97	246	[illegible]
220	135	135	257	267	153	159	148	148	113	113	145	147	91	97	240	[illegible]
231	139	139	259	263	153	153	148	158	119	119	145	155	91	91	240	[illegible]
235	129	139	263	269	153	153	158	160	113	127	145	155	91	91	240	[illegible]
241	129	131	257	257	149	153	146	146	107	109	145	145	91	91	240	[illegible]
243	135	139	257	259	145	155	148	160	113	115	145	145	97	97	246	[illegible]
248	131	135	257	269	145	159	160	160	109	109	147	155	97	97	240	[illegible]
252	129	129	259	269	149	173	158	158	121	121	145	145	93	97	236	[illegible]
257	131	139	267	271	153	175	148	160	113	113	145	145	91	91	240	[illegible]
261	129	139	269	269	145	163	160	160	107	109	145	145	91	97	240	[illegible]
300	131	135	263	267	153	179	146	148	113	115	145	159	97	97	256	[illegible]
A1_317	129	135	267	273	149	149	160	160	107	113	145	147	91	93	248	[illegible]
A10_028	129	135	263	269	163	173	160	162	113	115	145	145	95	97	256	[illegible]
A11_028	131	131	259	259	153	153	156	160	113	121	145	155	91	95	240	[illegible]
A12_028	129	135	259	267	165	179	148	160	115	115	145	155	91	93	256	[illegible]
A13_028	131	135	281	281	149	153	158	160	113	121	145	145	93	95	240	[illegible]
A14_028	135	135	273	281	149	173	156	160	121	127	145	145	91	91	256	[illegible]
A15_028	129	135	269	269	173	173	160	160	115	115	145	145	97	97	246	[illegible]
A16_028	133	139	269	279	165	173	160	160	107	121	145	145	91	91	248	[illegible]
A2_317	131	135	259	267	165	165	158	160	113	115	145	145	91	97	240	[illegible]
A20_038	129	131	259	273	149	179	160	160	113	113	145	147	91	91	246	[illegible]
A21_038	129	135	259	259	149	149	160	168	107	107	145	145	97	97	250	[illegible]
A22_038	129	131	263	273	149	153	158	166	107	107	145	147	95	97	256	[illegible]
A25_078	131	135	259	269	173	179	156	160	113	115	145	145	91	97	246	[illegible]
A27_078	131	135	263	267	163	163	160	160	115	127	145	145	93	97	246	[illegible]
A28_078	131	133	263	269	153	173	148	156	107	115	145	145	93	97	246	[illegible]
A3_317	135	139	267	267	159	163	148	160	107	115	145	155	91	91	274	[illegible]
A30_078	139	139	273	273	173	173	160	160	107	113	145	145	95	97	240	[illegible]
A31_078	131	133	269	273	173	173	148	160	107	113	145	145	97	97	246	[illegible]

A32_078	131	131	267	273	173	173	156	160	113	115	145	145	91	97	246	[illegible]
A34_078	135	139	267	269	163	173	156	158	113	115	145	145	97	97	278	[illegible]
A35_088	135	139	267	267	159	163	160	160	113	113	145	155	91	91	246	[illegible]
A37_088	131	135	259	269	173	179	156	160	113	115	145	145	91	97	246	[illegible]
A4_317	137	137	0	0	149	149	148	148	123	125	145	145	91	91	246	[illegible]
A43_088	131	131	271	273	0	0	156	160	113	113	145	145	97	97	274	[illegible]
A47_098	131	139	269	273	173	173	160	160	107	127	145	145	97	97	240	[illegible]
A5_317	129	135	259	269	153	173	148	160	113	115	145	145	93	97	240	[illegible]
A56_098	131	135	271	273	173	179	160	168	109	113	155	155	91	91	256	[illegible]
A59_108	135	135	269	269	173	173	158	160	115	115	145	145	97	97	0	0
A61_108	131	139	267	267	159	163	152	156	107	113	145	145	91	91	256	[illegible]
A62_108	129	135	259	281	149	165	148	162	113	115	145	145	91	91	248	[illegible]
A68_108	139	139	257	267	163	163	160	160	113	113	145	145	91	91	246	[illegible]
A9_028	131	131	267	267	165	165	160	160	127	127	145	145	93	93	256	[illegible]

Missing values are indicated by “0”.

Table S3
Unique genotypes (*N* = 65) of unrelated (r <0.5) hedgehogs in Berlin across ten microsatellite loci

ID	EEU1		EEU2		EEU3		EEU4		EEU5		EEU6		EEU12H		EEU36	
110	135	139	257	257	145	153	148	148	113	129	145	145	91	97	246	[illegible]
127	135	135	257	257	145	163	160	160	113	135	145	145	95	97	240	[illegible]
129	135	143	259	267	149	163	160	170	113	135	145	155	91	97	236	[illegible]
135	135	139	259	279	149	153	156	158	113	113	145	145	91	91	246	[illegible]
139	131	135	269	269	145	155	144	158	109	113	145	145	91	97	240	[illegible]
140	135	139	267	281	145	153	148	148	115	129	145	145	91	97	240	[illegible]
184	129	131	267	269	145	145	154	160	109	129	145	145	91	91	240	[illegible]
185	135	137	269	271	153	169	146	152	109	113	145	159	91	97	256	[illegible]
188	129	139	257	269	153	179	160	170	113	129	145	145	91	95	240	[illegible]
191	129	129	267	267	153	169	152	152	113	113	145	145	91	97	256	[illegible]
192	135	139	257	259	159	179	146	160	107	113	145	147	91	97	246	[illegible]
193	129	139	269	277	169	169	160	164	113	113	145	145	91	97	240	[illegible]
134	135	139	267	275	149	163	148	150	113	115	145	145	95	97	240	[illegible]
194	139	139	257	267	145	159	148	160	113	123	145	145	97	97	240	[illegible]
196	139	139	259	267	145	179	148	160	107	123	145	145	91	97	238	[illegible]
198	129	137	259	267	163	165	148	158	109	113	145	145	97	97	248	[illegible]
199	131	131	257	257	153	153	148	148	113	125	145	145	91	91	250	[illegible]
114	131	139	259	269	149	153	158	160	113	113	145	145	91	97	240	[illegible]
116	135	135	263	265	159	177	148	160	113	129	145	145	95	97	238	[illegible]
117	135	139	267	271	145	169	150	170	113	123	145	145	97	97	248	[illegible]
119	131	139	263	273	145	149	152	160	113	139	145	145	91	95	250	[illegible]
120	135	139	269	281	149	169	148	160	115	139	145	147	91	91	236	[illegible]
143	129	129	257	259	153	159	150	150	109	113	145	145	91	97	240	[illegible]
150	131	135	257	265	159	163	158	164	115	121	145	145	97	97	256	[illegible]
152	139	139	269	269	153	153	148	148	113	113	145	145	97	97	240	[illegible]
153	135	139	267	269	145	163	156	158	113	113	145	155	91	95	240	[illegible]
157	129	131	259	267	145	179	158	158	115	115	145	153	91	95	248	[illegible]
158	135	135	273	275	149	165	166	170	113	113	145	155	95	97	248	[illegible]
159	135	139	275	275	149	165	148	160	115	129	145	145	91	91	248	[illegible]
161	129	139	267	279	149	153	158	158	113	113	145	155	91	91	236	[illegible]

168	139	139	267	269	145	165	158	158	113	115	145	155	91	91	236	[illegible]
169	131	135	257	257	145	145	158	170	113	121	145	145	91	91	248	[illegible]
170	131	135	269	269	145	153	158	160	113	113	145	155	95	97	248	[illegible]
172	135	139	261	269	145	163	150	160	115	129	145	147	91	95	240	[illegible]
324	135	139	271	273	149	163	148	160	113	115	145	147	91	97	240	[illegible]
333	139	141	257	267	153	163	148	160	113	129	145	145	91	97	240	[illegible]
334	133	133	265	271	145	159	148	170	113	113	145	145	91	97	256	[illegible]
337	139	139	257	259	153	159	148	158	107	113	145	159	95	95	240	[illegible]
338	131	143	259	265	145	153	150	158	113	115	145	145	91	97	240	[illegible]
340	139	139	267	267	163	165	148	158	113	115	145	155	91	95	248	[illegible]
341	133	139	257	269	145	171	158	164	107	113	145	145	91	97	240	[illegible]
113	135	139	257	269	153	171	148	160	113	131	145	145	91	91	244	[illegible]
118	133	139	257	267	145	145	160	164	113	129	145	155	97	97	240	[illegible]
309	135	139	267	271	145	153	160	160	113	123	145	145	91	91	256	[illegible]
310	129	135	269	273	145	163	148	160	113	113	145	145	91	97	248	[illegible]
343	129	131	267	267	131	179	156	158	115	115	145	145	91	97	246	[illegible]
231	139	139	259	263	153	153	148	158	119	119	145	155	91	91	240	[illegible]
235	129	139	263	269	153	153	158	160	113	127	145	155	91	91	240	[illegible]
241	129	131	257	257	149	153	146	146	107	109	145	145	91	91	240	[illegible]
243	135	139	257	259	145	155	148	160	113	115	145	145	97	97	246	[illegible]
248	131	135	257	269	145	159	160	160	109	109	147	155	97	97	240	[illegible]
252	129	129	259	269	149	173	158	158	121	121	145	145	93	97	236	[illegible]
300	131	135	263	267	153	179	146	148	113	115	145	159	97	97	256	[illegible]
A10_028	129	135	263	269	163	173	160	162	113	115	145	145	95	97	256	[illegible]
A11_028	131	131	259	259	153	153	156	160	113	121	145	155	91	95	240	[illegible]
A12_028	129	135	259	267	165	179	148	160	115	115	145	155	91	93	256	[illegible]
A13_028	131	135	281	281	149	153	158	160	113	121	145	145	93	95	240	[illegible]
A14_028	135	135	273	281	149	173	156	160	121	127	145	145	91	91	256	[illegible]
A20_038	129	131	259	273	149	179	160	160	113	113	145	147	91	91	246	[illegible]
A21_038	129	135	259	259	149	149	160	168	107	107	145	145	97	97	250	[illegible]
A22_038	129	131	263	273	149	153	158	166	107	107	145	147	95	97	256	[illegible]
A3_317	135	139	267	267	159	163	148	160	107	115	145	155	91	91	274	[illegible]
A4_317	137	137	0	0	149	149	148	148	123	125	145	145	91	91	246	[illegible]

A61_108	131	139	267	267	159	163	152	156	107	113	145	145	91	91	256	2[illegible]
A62_108	129	135	259	281	149	165	148	162	113	115	145	145	91	91	248	2[illegible]
